# Supplementary material for: Stage-aware transcriptomics reveals selective haplotype persistence in short-term ex vivo cultured Plasmodium vivax
Source: bioRxiv. 2026 May 13:2026.05.11.724466. Preprint. [Version 1] doi: 10.64898/2026.05.11.724466 (PMC13192822; doi:10.64898/2026.05.11.724466)
Supplement: 1 [file NIHPP2026.05.11.724466V1-supplement-1.pdf]

bioRxiv preprint doi: https://doi.org/10.64898/2026.05.11.724466; this version posted May 13, 2026. The copyright holder for this preprint (which was not certified by peer review) is the author/funder, who has granted bioRxiv a license to display the preprint in perpetuity. It is made available under a CC-BY 4.0 International license.

Supporting Information

bioRxiv preprint doi: https://doi.org/10.64898/2026.05.11.724466; this version posted May 13, 2026. The copyright holder for this preprint (which was not certified by peer review) is the author/funder, who has granted bioRxiv a license to display the preprint in perpetuity. It is made available under a CC-BY 4.0 International license.

Supplementary Figure

S1A-B Fig: Unsupervised principal component analysis (PCA) revealed substantial overlap between *in vivo* and short-term cultured samples when colored by experimental condition. However, samples stratified by schizont stage fraction exhibited stage-associated clustering patterns, indicating that developmental composition is a primary driver of transcriptional variance. 1**C-D:** Following adjustment for parasite developmental stage, PCA showed no distinct clustering by Schizont stage composition.

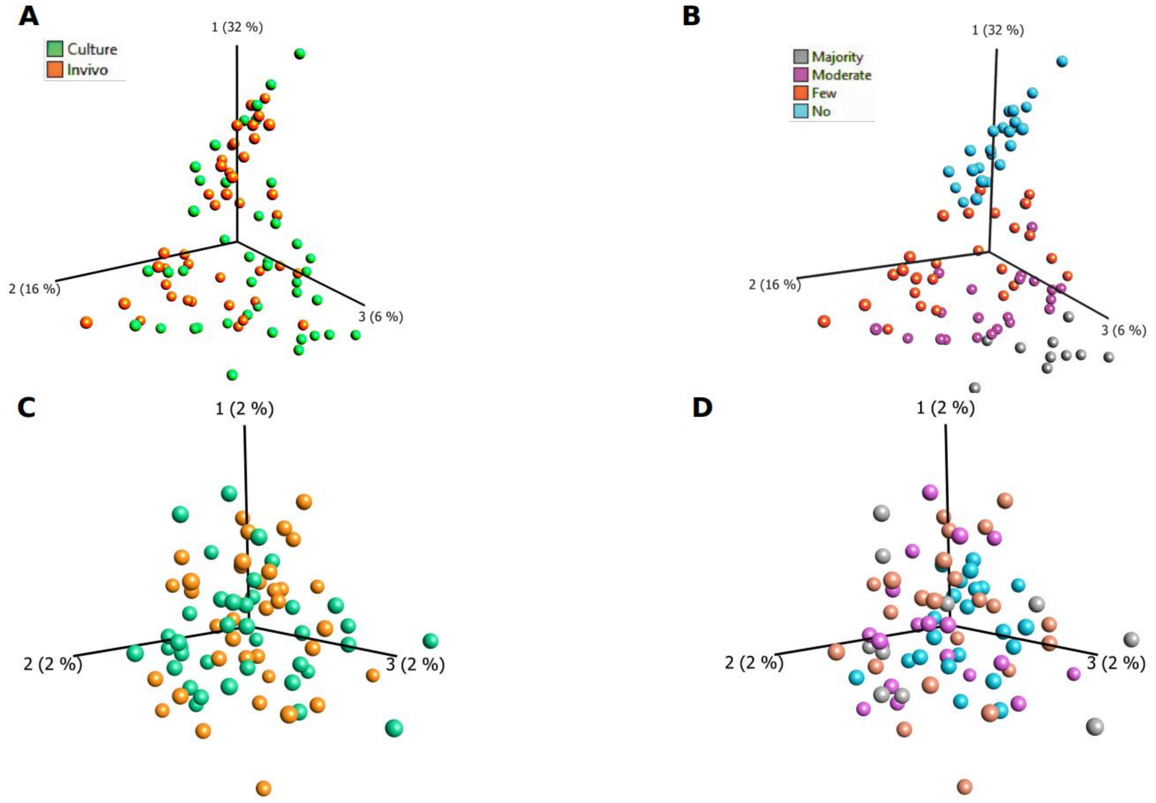

Supplementary Tables

S1 Table. Clinical and sequencing metadata for all analyzed samples including sample ID, infection source, culture condition, dominant haplotype, MOI classification, parasitemia category, and host Duffy genotype.

bioRxiv preprint doi: https://doi.org/10.64898/2026.05.11.724466; this version posted May 13, 2026. The copyright holder for this preprint (which was not certified by peer review) is the author/funder, who has granted bioRxiv a license to display the preprint in perpetuity. It is made available under a CC-BY 4.0 International license.

S2 Table. Parasite developmental stage fractions.

Estimated stage proportions for each sample including ring, trophozoite, schizont, and gametocyte fractions.

S3 Table. Description of the SRA submission

Supplementary Data

S1 Data. Raw gene expression matrix used for transcriptomic analyses.

S2 Data. Stage-adjusted residual expression values used for PCA, heatmap and Venn diagram.

S3 Data. Haplotype-level summary statistics for net haplotype change and enrichment analysis.
